# Activation and Inhibition of The *Wnt3A* Signaling Pathway in Buffalo (*Bubalus bubalis*) Embryonic Stem Cells: Effects of WNT3A, Bio and Dkk1

**DOI:** 10.22074/ijfs.2015.4552

**Published:** 2015-10-31

**Authors:** Mohammad Zandi, Syed Mohamad Shah, Musharifa Muzaffar, Manoj Kumar Singh, Prabhat Palta, Suresh Kumar Singla, Radhey Sham Manik, Manmohan Singh Chauhan

**Affiliations:** 1 Department of Animal and Poultry Science and Fisheries, Agricultural Institute, Iranian Research Organisation for Science and Technology (IROST), Tehran, Iran; 2 Embryo Biotechnology Laboratory, Animal Biotechnology Centre, National Dairy Research Institute (NDRI), Karnal, India

**Keywords:** WNT3A, Buffalo, Embryonic Stem Cells, Bio, Dkk1

## Abstract

**Background:**

This research studies the effects of activation and inhibition of *Wnt3A* signaling pathway in buffalo (*Bubalus bubalis*) embryonic stem (ES) cell-like cells.

**Materials and Methods:**

To carry on this experimental study, the effects of activation and inhibition of *Wnt3A* signaling in buffalo ES cell-like cells were examined using Bio (0.5 mM) combined with WNT3A (200 ng/ml), as an activator, and Dickkopf-1 (Dkk1, 250 ng/ml), as an inhibitor, of the pathway. ES cells were cultured up to three weeks in ES cell medium without fibroblast growth factor-2 (FGF-2) and leukemia inhibitory factor (LIF), but in the presence of Bio, WNT3A, Bio+WNT3A and Dkk1. The effects of these supplements were measured on the mean area of ES cell colonies and on the expression levels of a number of important genes related to pluripotency (*Oct4, Nanog, Sox2* and *c-Myc*) and the *Wnt* pathway (*β-catenin*). ES cell colonies cultured in ES cell medium that contained optimized quantities of LIF and FGF-2 were used as the control. Data were collected for week-1 and week-3 treated cultures. In addition, WNT3A-transfected ES cells were compared with the respective mock-transfected colonies, either alone or in combination with Dkk1 for expression of *β-catenin* and the pluripotency-related genes. Data were analyzed by ANOVA, and statistical significance was accepted at P<0.05.

**Results:**

Among various examined concentrations of Bio (0.5-5 mM), the optimum effect was observed at the 0.5 mM dose as indicated by colony area and expressions of pluripotency-related genes at both weeks-1 and -3 culture periods. At this concentration,the expressions of *Nanog, Oct3/4, Sox2, c-Myc* and *β-catenin* genes were nonsignificantly higher compared to the controls. Expressions of these genes were highest in the Bio+WNT3A treated group, followed by the WNT3A and Bio-supplemented groups, and lowest in the Dkk1-treated group. The WNT-transfected colonies showed higher expressions compared to both mock and Dkk1-treated mock transfected colonies.

**Conclusion:**

WNT3A functions to maintain the pluripotency of ES cell-like cells both as an exogenous growth factor as well as an endogenously expressed gene. It complements the absence of FGF-2 and LIF, otherwise propounded essential for buffalo ES cell culture. WNT3A antagonizes the inhibitory effects of Dkk1 and acts in combination with its activator, Bio, to activate the *Wnt* signaling pathway.

## Introduction

Buffalo embryonic stem ( ES ) cell-like cells are derived from the inner cell mass of the blastocysts and can be maintained in culture conditions that retain their pluripotency. Among the various intrinsic and extrinsic factors that maintain ES cell pluripotency, leukemia inhibitory factor ( LIF ) and fibroblast growth factor-2 ( FGF-2 ) are well characterized (1). These are required either alone, for example LIF in case of mouse ES cells and FGF-2 in human ES cells, or in combination as in the case of buffalo ES cells. 

Though feeder-dependent cultures are presumed to be better for long-term cultures, conditioned media ( cm ) from mouse embryonic fibroblast ( MEF ) can support the self-renewal of ES cells and eliminate the need for a feeder layer. It has been demonstrated that MEF inhibits ES cell differentiation via production of the IL-6 family cytokine and LIF (2). LIF binds the heterodimeric LIF receptor-glycoprotein 130 ( gp130 ) complex and activates Jak kinases with recruitment of *Shp-2* and signal transducer and activator of transcription 3 ( STAT3 ) (3) while FGF-2 signals are transduced through receptors with intrinsic protein tyrosine kinase activity (4,6). 

FGF-2 supplementation is associated with pleiotropic-positive effects: impeding spontaneous differentiation, increasing human ES cell proliferation, enhancing attachment/survival, inhibiting earliest neural induction, and, more precisely, moderately stimulating *Nanog* gene expression. In contrast, the FGF/ERK cascade plays a role in the differentiation of mouse ES cells (7). Since increased telomerase activity is presumed to be pivotal for ES cell self-renewal, the study of the pathways that control telomerase activity has gained a considerable interest in stem cell studies. Among the various studies reported so far in this context, a molecular link between *Wnt/β-catenin* signaling and the expression of the telomerase subunit *Tert* has gained a considerable interest owing to contrasting associations of *Wnt* signaling with both proliferation and differentiation of ES cells. 

WNT genes, of which the human genome harbors almost 20, occur throughout the animal kingdom (8). The proteins constitute a family of cysteinerich secreted ligands essential for a wide array of developmental and physiological processes. 

The intracellular signaling pathway activated by WNT has been originally identified as a β-catenindependent pathway that is highly conserved among various species. WNTs act through the cytoplasmic protein Dishevelled ( Dsh ) to inhibit the activity of the serine-threonine kinase, GSK3-β, which otherwise bind to the β-catenin-APC complex through Axin, leading to β-catenin phosphorylation and rapid degradation. WNT-induced inhibition of GSK3-β causes β-catenin stabilization which results in its increased level in the uncomplexed soluble form. This latter form can interact with TCF/ LEF transcription factors and, after translocation to the nucleus, activate target genes such as *Myc, CyclinD1, Axin2* and *Siamois*. Most of these genes have one or more TCF-binding elements near the transcription start site in their promoter region and play roles in the regulation of gene expression, cell proliferation, differentiation, and maintenance of cell polarity (9,10). 

*Wnt* signaling has been shown to play a role in the regulation of self-renewal of both mouse and human ES cells independently of LIF/STAT3 signaling. It is associated with both proliferation and differentiation of ES cells and therefore, the role of *Wnt* signaling in ES cells remains controversial (11). 

Sato et al. (12) have found that *Wnt* pathway activation by Bio, a specific pharmacological inhibitor of GSK3-β, maintains the undifferentiated phenotype in both types of ES cells and sustains expression of the pluripotent state-specific transcription factors such as *Oct3/4, Rex-1* and *Nanog* (13). Hence, low GSK3 activity could be an absolute requirement for pluripotency and ES cell selfrenewal (14). Using a high-throughput cell-based assay, Miyabayashi et al. (11) have identified the small molecule Iq-1 that allows for *Wnt/β-catenin* driven long-term expansion of mouse ES cells and prevention of spontaneous differentiation. 

In addition to the GSK3-β/Axin/APC destruction complex, the *Wnt* pathway is also controlled by extracellular antagonists such as Wnt inhibitory signaling factor-1 ( WIF1 ), Cerebrus, Sclerostin, Dickkopf-1 ( Dkk1 ) and SFRP2 (15). Cerebrus, WIF1 and SFRP2 interact directly with WNT proteins, however Sclerostin and Dkk1 bind to LRP5/6 and indirectly exert their antagonizing effects (16). Different Frizzled-related protein and Dkk family members have shown opposite effects in a variety of *in vivo* and *in vitro* assays (17). 

In order to investigate the effects of *Wnt* signaling on ES cells, the present study was designed to examine the effects of *Wnt3A* signaling activation on buffalo ( *Bubalus bubalis* ) ES cell-like cells, which would provide a higher mammalian model, by addition of Bio as the activator. To ensure that the effects are due to activation of *Wnt* signaling pathway, we used Dkk1 as the pathway inhibitor to examine the contrary effects and Wnt 3A, in both exogenous and endogenous forms, to corroborate the primary results. 

## Materials and Methods

### Chemicals

To carry on this experimental study, unless mentioned otherwise, all culture media, growth factors, fetal bovine serum ( FBS ), Bio ( B1686 ) and other chemicals were purchased from Sigma ( USA ) and plastic ware was purchased from Falcon ( UK ). Recombinant human WNT3A ( 5036-wn ) and recombinant human Dkk1 ( 5439-DK ) were purchased from R and D systems. 

### In vitro embryo production

Buffalo ovaries were obtained from a local abattoir and transported to the laboratory in phosphatebuffered saline that contained penicillin (100 IU/ mL) and streptomycin (50 mg/mL) at 30-34˚C within 5 hours of slaughter. Cumulus-oocyte complexes (COCs), from follicles 2-8 mm in diameter, were aspirated using an 18 G needle attached to a 10 mL disposable syringe. A group of 15 to 20 excellent quality COCs were transferred to a 100 mL droplet of the *in vitro* maturation (IVM) medium [TCM 199+10% FBS+5 μg/mL porcine follicle stimulating hormone (pFSH)+1 μg/mL estradiol- 17 β+0.81 mM sodium pyruvate+5-10% buffalo follicular fluid+50 μg/mL gentamicin sulfate] under mineral oil in a petri dish and cultured at 38.5˚C in a humidified atmosphere of 5% CO_2_ for 24 hours. The *in vitro* matured (IVM) oocytes were washed twice with Bracket and Oliphant’s (BO) medium and transferred to 50 μL droplets (15-20 oocytes/droplet) of the medium. The spermatozoa were prepared for fertilization as per the protocol established by Chauhan et al. (18). Oocytes were then inseminated by addition of spermatozoa at a final concentration of 1.0-2.0×10^6^ motile sperm/ mL. Sperm and oocytes were incubated under paraffin oil at 38.5˚C under a humidified atmosphere of 5% CO_2_ for 18 hours. At the end of the interval, groups of 10 oocytes stripped free from cumulus cells were transferred into modified Charles Rosenkrans medium with amino acids (mCR2aa) that contained 0.6% bovine serum albumin (BSA) and cultured in this medium for the first 2 days, which was then replaced by *in vitro* culture (IVC) medium (mCR2aa+0.6% BSA+10% FBS). The culture medium was changed every 2 days up to 8 days until the blastocysts were obtained.

### Establishment of buffalo embryonic stem cells

Buffalo ES cells were derived from the *in vitro* fertilized embryos as described by Muzaffar et al. (19). Briefly, we mechanically dissected the inner cell mass from the embryos under a zoom stereomicroscope and seeded them onto a mitomycintreated buffalo fetal fibroblast feeder layer in ES medium that consisted of knockout Dulbecco’s modified eagle medium ( KO-DMEM, Gibco/ BRL ) supplemented with 15% knockout serum replacement medium ( Gibco/BRL ), 2 mM L-glutamine, 0.1 mM β-mercaptoethanol, 1% nonessential amino acids ( all from Gibco/BRL ), 1000 U/ mL LIF, and 5 ng/mL FGF-2 ( R & D Systems ). The media was changed every alternate day and the resultant colonies were sub-cultured onto fresh feeders after every 7 days. LIF and FGF-2 were added only to the control group media while the treatment group media lacked these factors. Bio, WNT3A, and Dkk1 were added to the latter media at every media change at their optimized concentrations. 

### Characterization of the embryonic stem cells

**Fig.1:**
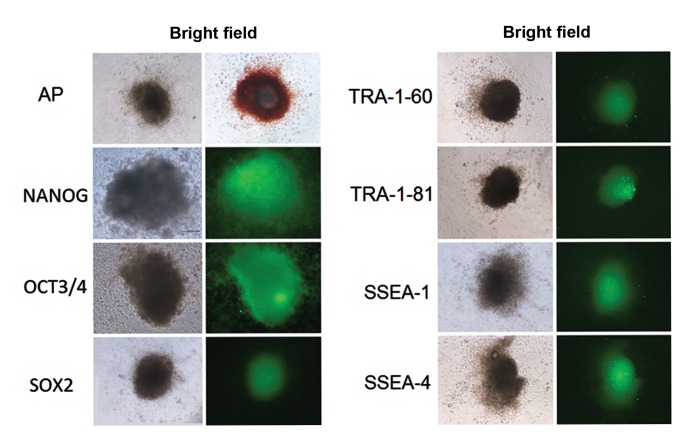
Alkaline phosphatase (AP) and immunofluorescence staining for characterization of buffalo embryonic stem (ES) cells at passage 20. Cell surface antigens SSEA-1 and SSEA-4, keratan sulfate antigens TRA-1-60 and TRA-1-81, and pluripotency markers Nanog, Oct3/4 and Sox2 were used to characterize ES cells.

Alkaline phosphatase ( AP ) staining and immunofluorescence were used for characterization of buffalo ES cells, as per a previously described protocol (20). The cell surface antigens used for characterization comprised glycolipids: SSEA-1 and SSEA-4; keratan sulfate antigens: TRA-1-60 and TRA-1-81 ( Chemicon, Millipore, Cat. No. SCR002) and the pluripotency markers Nanog ( Santa Cruz, Cat. No. SC 134218 ), Oct3/4 ( Chemicon, Millipore, Cat. No. SCR002 ) and Sox2 ( Chemicon, Millipore, Cat. No. SC1002 ). We used a 1:50 dilution of primary antibodies while the appropriate flourescein isothiocyante ( FITC )-conjugated secondary antibodies were diluted 1:500 in Dulbecco’s phosphate-buffered saline (Fig .1). 

Cell surface antigens SSEA-1 and SSEA-4, keratan sulfate antigens TRA-1-60 and TRA-1-81, and pluripotency markers *Nanog, Oct3/4* and Sox2 were used to characterize ES cells. 

### Estimation of colony area

An inverted microscope ( Nikon, Japan, Model Eclipse Ti 5 ), equipped with the software for calculation of the colony area was used to estimate the colony area. 

### RNA isolation, reverse transcription and quantitative real-time PCR (qPCR)

Total RNA was isolated with the Trizol reagent ( Invitrogen ) and subsequently treated with DNAse ( Ambion, USA ) to eliminate DNA contamination. Reverse transcription was performed with MMLV enzyme ( USB ) using oligo dT primers. qPCR was carried out with SYBR Green mix ( ABI ). Calculations were based on the ∆∆Ct method taking GAPDH as the endogenous control. Primer sequences are listed in table 1. 

### Cloning of the *Wnt3A* gene

Primers were designed from *Bos taurus* full length *Wnt3A* gene sequence ( Accession No. XM_002688509.1) using Primer 3 Software. The polymerase chain reaction ( PCR ) cycling conditions were 94˚C for 3 minutes, followed by a cycling program of 94˚C for 30 seconds, 60˚C for 45 seconds and 72˚C for 30 seconds for 36 cycles, and a final extension at 72˚C for 10 minutes. The amplified *Wnt3A* gene was analyzed on a 2% agarose gel. The PCR product was purified from the gel as per the manufacturer’s protocol using the QIAquick Gel Extraction Kit ( Cat. No. 28704) and ligated into a pDrive cloning vector according to the manufacturer’s guidelines ( Qiagen PCR Cloning Kit ). The ligated product that equaled 5% of the cell volume was added to competent cells ( *E.coli XL1* ), the contents mixed and kept on ice for 30 minutes. Bacterial transfection was achieved by heat shock at 42˚C for 90 seconds, then chilled on ice for 2 minutes. Next, 800 µl of super optimal broth ( SOC ) was added to each tube followed by incubation in a shaking incubator at 37˚C for 42 minutes at 225 cycles/minute. The cells were then centrifuged at 3000 rpm for 3 minutes and the pellet was plated on an Luria Bertani ( LB ) agar plate for overnight incubation at 37˚C. The colonies which had the insert were verified by colony PCR and restriction enzyme test digestion. The positive clones were propagated and subjected to plasmid isolation for sequencing of the insert. The insert in the correct reading frame was subsequently ligated into a pAcGFP1-N1 vector, as a GFP-fusion protein, employing EcoR1 and Bam H1 restriction sites for generation of cohesive ends. The ligation reaction was prepared as: vector ( 20-100 ng ), insert DNA ( 3:1 to 5:1 molar ratio with vector ), 10X T4 DNA ligase buffer ( 2 μL ) and T4 DNA ligase ( 1 μL ), and incubated for 10 minutes at 22˚C. 

### Overexpression of Wnt3A in buffalo embryonic stem cell-like cell colonies

**Fig.2:**
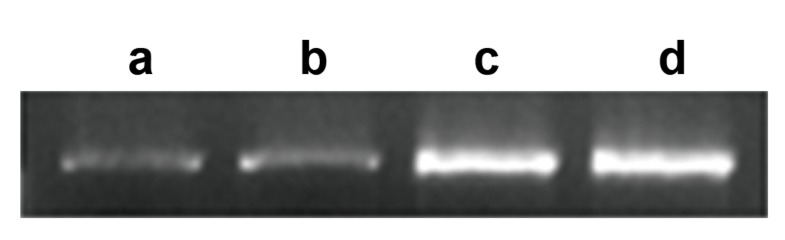
RT-PCR of transfected and non-transfected colonies. Agarose gel electrophoresis for Wnt3A expression (cDNA). Lane a; Control, Lane b; MOCK vector transfected colonies, Lanes c, d; Wnt3A transfected colonies and RT-PCR; Real-time polymerase chain reaction.

ES cell colonies were mechanically divided into small parts, then cultured in 100 μL droplets of the ES cell culture medium for three days prior to transfection. The vector that contained the insert (pAcGFP1-N1+*WNT3A*, 2-5 μg) was diluted in 50 μL DMEM without serum and mixed with Lipofectamine™ 2000 (Invitrogen) also diluted in 50 μL of the medium. The mixture was incubated for 20 minutes at room temperature and used to replace the ES cell culture medium in which the ES cell colonies were growing. The cells were incubated at 37˚C in a CO_2_ incubator for 18-48 hours, with daily media changes until GFP expression was evident. The colonies that expressed GFP were separated individually and used for further studies. The expression of Wnt3A in transfected colonies was further confirmed by RT-PCR (Fig.2).

**Table 1:** Real-time polymerase chain reaction (PCR) primers


Gene	Sequence	Annealingtemp.(˚C)	Basepairs	Accession no.

*Sox2*	F: 5΄CGTGGTTACCTCTTCTTCC3΄	60	139	GQ85388
	R:5΄CTGGTAGTGCTGGGACAT3΄			
*Oct3/4*	F: 5΄TTGCAGCTCAGTTTCAAG3΄	54	75	EU926737
	R:5΄GTTGTTGTCAGCTTCCTC3΄			
*Nanog*	F: 5΄CCGAAGCATCCAACTCTAGG3΄	60	100	NM001025344.1
	R:5΄GAGACAGTGTCCGTGTCGAG3΄			
*c-Myc*	F: 5΄CTCCTCACAGCCCGTTAGTC3΄	53	156	GU296437.1
	R:5΄ATTTGCGGTTGTTGCCTATC3΄			
*β-catenin*	F: 5΄ACAGAAAAGCAGCCGTCAGT3΄	56	191	NM001076141.1
	R:5΄AGAAAACCCCTGTTCCCACT3΄			
*GAPDH*	F: 5΄TCAAGAAGGTGGTGAAGCAG3΄	57	121	GU324291.1
	R:5΄CCCAGCATCGAAGGTAGAAG3΄			


### Experimental design

#### Experiment 1: Optimization of bio concentrations for buffalo embryonic stem cell culture

This experiment was performed with five doses to optimize the Bio concentration: 1. ES cell medium as a control, 2. ES cell medium+0.5 μM Bio, 3. ES cell medium+1 μM Bio, 4. ES cell medium+2 μM Bio and 5. ES cell medium+5 μM Bio. 

#### Experiment 2: Effects of activation and inhibition of the *Wnt3A* signaling pathway on buffalo embryonic stem cells

#####  Experiment 2.1

This experiment was performed to examine the effects of exogenous WNT3A and Wnt pathway activator and inhibitor on ES cell growth and stemness in1 and 3 week cultures. The treatments were as follows: 1. ES cell medium as a control, 2. ES cell medium+0.5 μM Bio, 3. ES cell medium+0.5 μM Bio+200 ng/ml WNT3A, 4. ES cell medium+250 ng/ml Dkk1 and 5. ES cell medium+200 ng/ml WNT3A.

##### Experiment 2.2

This experiment was performed to examine the effects of overexpression of WNT and its ability to counteract the inhibitory effects of Dkk1 on ES cell pluripotency and the *Wnt* pathway by comparing expression of pluripotency maintaining genes and β-catenin, respectively. The treatments were as follows 1. MOCK vector-transfected ES cells ( control ), 2. WNT3A-transfected ES cells and 3. Mock vector-transfected ES cells+Dkk1 250 ng/ml. 

### Statistical analysis

Data were analyzed with a statistical software program ( SPSS 11.5, 2004 ). Comparisons between multiple numeric data sets were performed using one-way ANOVA followed by Duncan’s multiple range test. 

The results were expressed as mean ± SEM and statistical significance was accepted at P<0.05. 

## Results

### Effect of Bio on pluripotency and Wnt signaling

**Fig.3:**
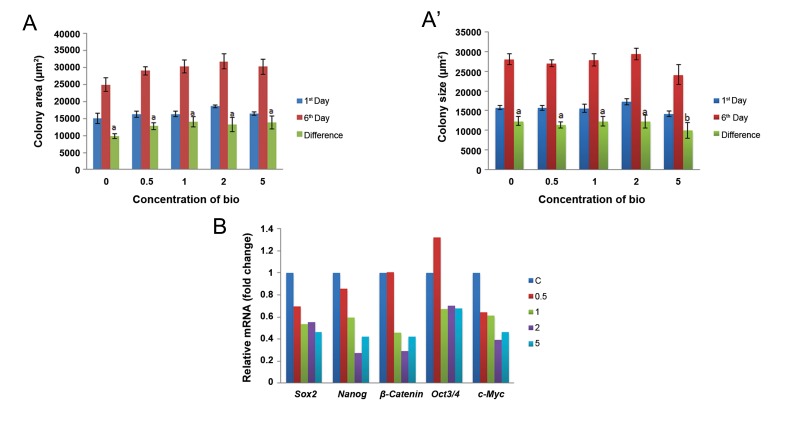
The effects of different concentrations of Bio (μM). Mean area of buffalo embryonic stem (ES) cell colonies at: A. First week of exposure. A΄. Third week of exposure and B. Effect on expression of β-catenin and pluripotency genes after three weeks of exposure. Bars with different superscripts differ significantly while those with the same superscripts do not significantly differ at P<005.

The ES cells exposed to Bio, in a dose-and time-dependent manner, showed optimum activity at a concentration of 0.5 μM for day 1 versus day 6 of week 1 as well as for day 1 versus day 6 of week 3 on cell proliferation ( mean colony area ), self-renewal ( expression of pluripotency genes ) and *Wnt* pathway activation ( *β-catenin* expression ). We observed no statistically significant difference in colony area at different concentrations of Bio ( 0.5, 1, 2 and 5 μM ) on days 1 and 6 of the first week of culture, although a trend towards increase in the colony area was observed which indicated a stimulatory effect of Bio on cellular proliferation (Fig .3A). A similar trend which indicated an increase in the colony area was observed between days 1 and 6 of the third week of culture, with the exception of the 5 μM Bio concentration when mean colony area was smaller, with a statistically significant difference in the area (Fig .3A΄). Real-time PCR analysis showed that the expressions of *β-catenin* and pluripotency genes were relatively higher at the 0.5 μM Bio concentration compared to the other doses. At this concentration of Bio, the treatment groups exhibited almost similar growth and stemness properties as the control group. *β-catenin* expression was highest at the 0.5 μM Bio concentration, equivalent to the control, while it showed a thorough decrease at the other concentrations (Fig .3B). 

### Effect of WNT3A (exogenous and endogenous), Bio and Dkk1 on embryonic stem cell growth and Wnt signaling pathway 

**Fig.4:**
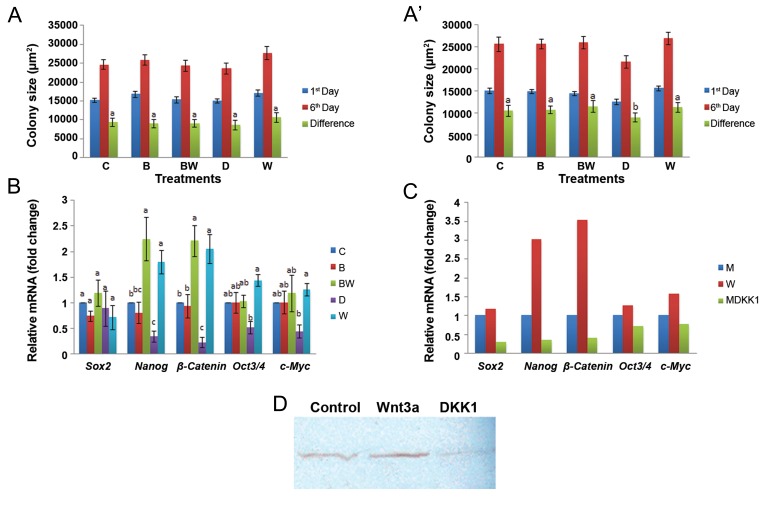
The effect of activation and inhibition of the *Wnt3A* signaling pathway. Mean area of buffalo embryonic stem (ES) cell colonies at: A. First week of exposure. A΄. Third week of exposure. B. Expression of β-catenin and pluripotency genes. C; Control, B; Bio, BW; Bio+WNT3A, D; Dkk-1 and W; WNT3A. C. Expression of β-catenin and pluripotency genes from transfected colonies (W; WNT3A transfected colonies, M; MOCK vector transfected colonies and MDkk1; MOCK vector transfected colonies+Dkk1) and D. Western blot analysis of buffalo ES cells for β-catenin (Control; ES medium, Wnt3a; ES medium+Wnt3a and Dkk1; ES medium+Dkk1). Bars with different superscripts differ significantly while those with the same superscripts do not significantly differ at P<005.

We used 200 ng/ml exogenous WNT3A optimized in our earlier study (20). In this experiment, the effects of Bio ( 0.5 μM ) combined with WNT3A ( 200 ng/ml ) for activation of the *Wnt3A* signaling pathway as well as Dkk1 ( 250 ng/ml ) for inhibition of the *Wnt3A* signaling pathway were studied on ES cell proliferation, *β-catenin* and pluripotency gene expression. Dkk1 treatment had no significant effect on mean colony area in the 1-week culture period ( day 1 vs. day 6 ) relative to the control where as a significant decrease was observed in the 3-week culture period ( day 1 vs. day 6, Fig.4A, A΄ ). Real-time PCR analysis showed decreased expressions of *Nanog, β-catenin, Oct3/4* and *c-Myc* genes in Dkk1 treated cultures in comparison to all other treatments viz. Bio alone, Bio+WNT and WNT alone (Fig .4B). WNT3A transfected colonies showed increased expression of all the genes under study compared to both the mock transfected colonies as well as Dkk1 treated mock transfected colonies (Fig .4C). The band that corresponded to the β-catenin protein was virtually absent in Dkk1 treated ES cell colonies when visualized parallel to the control and WNT3A treated colonies for detection of the protein (Fig .4D). 

## Discussion

Our results have shown that supplementation with Bio in the absence of LIF and FGF-2 can maintain ES cell growth and pluripotency in both weeks 1 and 3 culture periods. However, at higher concentrations ( 5 μM ), Bio adversely affected cellular proliferation in the 3-week culture period. The absence of a significant difference in the colony area between Bio-treated and feeder supported ( controls ) ES cell colonies may be due to the combined effects of Bio and feeder layer cells, since the feeder layer is thought to provide both LIF and FGF-2 (2).The equivalency in expression of *β-catenin* and pluripotency genes between the control and Bio-treatment group ( 0.5 μM ) indicates up-regulation of these genes by Bio treatment. The decrease in gene expression at higher concentrations of Bio can be due to probable toxicity or feedback inhibition of the *Wnt* signaling pathway or activation of other mechanisms of differentiation induction in ES cell colonies. This is in accordance with the report of Sato et al. (12) who have shown that *Wnt* pathway activation by Bio, a specific pharmacological inhibitor of GSK3, maintains the undifferentiated phenotype in both types of ES cells and sustains expression of the pluripotent state-specific transcription factors *Oct3/4, Rex-1* and *Nanog*. It has also been suggested that Bio may have a combinatorial effect on mouse ES cells, activating both the canonical Wnt signal and the LIF signal pathways simultaneously in order to maintain the cells in an undifferentiated state as in the case of the combination of recombinant WNT3A and LIF (21). 

**Fig.5:**
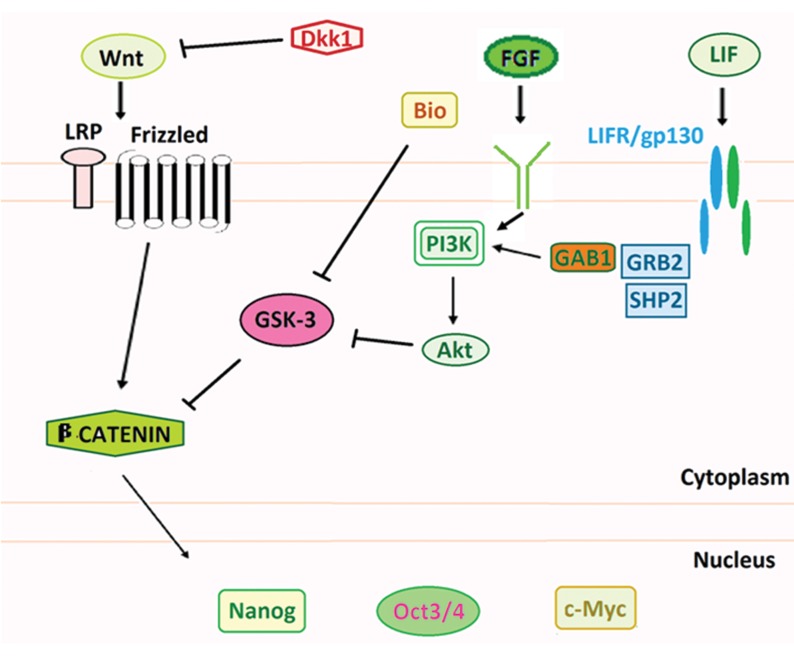
Predicted model for the action of GSK3-β in buffalo embryonic stem ( ES ) cells. Leukemia inhibitory factor ( LIF ) and fibroblast growth factor-2 ( FGF-2 ) stimulation appear to cause inactivation of GSK3-β through the PI3K/Akt pathway in buffalo ES cells. Hence, Bio, as a specific pharmacological inhibitor of GSK-3β, was unable to further inactive GSK3-β.

Doble et al. (22) have shown that the PI3K pathway, which is known to negatively regulate GSK3-β through serine phosphorylation of its amino-terminal domain, plays a role in the maintenance of mouse and primate ES cell pluripotency. Also, GSK3-β phosphorylation of *c-Myc* on *T58* has been implicated in the LIF/STAT3-mediated regulation of mouse ES cell pluripotency, since over expression of a *T58A* mutant of *c-Myc* in mouse ES cells promotes their self-renewal and pluripotency in the absence of exogenous LIF. Therefore, it seems that activation of the PI3K pathway by feeder layer secreting factors ( FGF2 or LIF ) can lead to inactivation of GSK3-β, in conjunction with exogenous Bio in our treatment groups. This enables the cells to retain their growth and pluripotency almost equivalent to FGF-2 and the LIF supplemented control group (Fig .5). 

It has also been predicted that suppression of GSK3-β activity by Bio, in the absence of *LIF/Wnt* signaling, can establish conditions where *c-Myc* is unphosphorylated on T58, leading to elevated *cMyc* levels and hence, enhanced stem cell stability and self renewal (23). 

Individual members of the Dkk family of secreted proteins could either antagonize or stimulate *Wnt* signaling through interaction with LRP6 (17). Our results showed that Dkk1 significantly ( P<0.05 ) decreased the growth of buffalo ES cells based on the mean area of colonies and expression of *β-catenin* and other pluropotency-related genes. In agreement with our result, Beildeck et al. (15) showed that Dkk family members and Wise, a context-dependent secreted protein, both antagonize the *Wnt* pathway by binding to LRP5/6 and preventing WNTs from binding, thereby executing their function to maintain stem cell growth and pluripotency in absence of exogenous FGF2 and LIF. It has also been reported that activation of the canonical *Wnt* signaling pathway could be measured by accumulation of *β-catenin* in the presence of the WNT protein (24). Sclerostin and other BMP antagonists had no effect on accumulation, whereas Dkk-1, a WNT inhibitor, completely blocked WNT-induced *β-catenin* accumulation and activation of the *Tcf/Lef* reporter gene (25). These findings together have supported the virtual absence of *β-catenin* in Dkk1 treated ES cell colonies. Dkk1 is a prototypic *Wnt* signaling inhibitor that binds to and antagonizes the function of LRP6 (26). In addition, Dkk1 has also been shown to antagonize LRP6 function via LRP6 degradation with or without clathrindependent internalization (26,27), thereby suppressing the *β-catenin* pathway. Thus both phosphorylation and internalization of LRP6 seem necessary to induce *β-catenin* accumulation. WNT3A exerts its antagonistic action against Dkk1 by binding to LRP6 and linking it to molecules that reside in lipid rafts and prevents its clathrin-mediated internalization, a prerequisite for its activity (27). 

Our real-time PCR analysis showed that *Nanog, β-catenin, Oct3/4* and *c-Myc* were downregulated in buffalo ES cell colonies when supplemented with Dkk1. It has been demonstrated that overexpression of Dkk-1 leads to down regulation of *c-Myc* and cyclin D1 expression, while reduction of Dkk-1 expression by RNA interference enabled upregulation of *β-catenin, c-Myc*, and *cyclin D1* in H7402 cells. It also promoted *β-catenin* translocation from the cytoplasm into the nuclei and increased cell migration (28). This would explain the reason behind decreased expression of pluripotency maintaining genes and smaller colony area in Dkk1 treated cells compared to other treatment groups. 

## Conclusion

In the absence of exogenous FGF-2 and LIF, activation of the *Wnt3A* signaling pathway with exogenous or endogenous WNT3A, either alone or in combination with an activator ( Bio ), could maintain pluripotency and growth of buffalo ES cells throughout the 3 week culture period. The decrease in ES cell colony area and expression level of pluripotency-related genes and *β-catenin*, in the presence of Dkk1 as a selective inhibitor of the *Wnt* signaling pathway, has indicated that the Wnt pathway complements FGF-2 and LIF, which are indispensable for ES cell self-renewal and pluripotency. 
